# Moving from rhetoric to reality: adapting Housing First for homeless individuals with mental illness from ethno-racial groups

**DOI:** 10.1186/1472-6963-12-345

**Published:** 2012-10-02

**Authors:** Vicky Stergiopoulos, Patricia O’Campo, Agnes Gozdzik, Jeyagobi Jeyaratnam, Simon Corneau, Aseefa Sarang, Stephen W Hwang

**Affiliations:** 1 Centre for Research on Inner City Health, The Keenan Research Centre in the Li Ka Shing Knowledge Institute of St. Michael’s Hospital, 30 Bond Street, Toronto, Ontario M5B 1 W8, Canada; 2 Department of Psychiatry, University of Toronto, 250 College Street, 8th Floor, Toronto, Ontario M5T 1R8, Canada; 3 Dalla Lana School of Public Health, University of Toronto, Health Sciences Building, 6th floor, 155 College Street, Toronto, Ontario, M5T 3 M7, Canada; 4 Sexology Department, Université du Québec à Montréal, Case postale 8888, succursale Centre-Ville, Montréal, Québec, H3C 3P8, Canada; 5 Across Boundaries: An Ethno-racial Mental Health Centre, 51 Clarkson Avenue, Toronto, Ontario, M6E 2 T5, Canada; 6 Division of General Internal Medicine, Department of Medicine, University of Toronto, Toronto, Ontario, Canada

## Abstract

**Background:**

The literature on interventions addressing the intersection of homelessness, mental illness and race is scant. The At Home/Chez Soi research demonstration project is a pragmatic field trial investigating a Housing First intervention for homeless individuals with mental illness in five cities across Canada. A unique focus at the Toronto site has been the development and implementation of a Housing First Ethno-Racial Intensive Case Management (HF ER-ICM) arm of the trial serving 100 homeless individuals with mental illness from ethno-racial groups. The HF ER-ICM program combines the Housing First approach with an anti-racism/anti-oppression framework of practice. This paper presents the findings of an early implementation and fidelity evaluation of the HF ER-ICM program, supplemented by participant narrative interviews to inform our understanding of the HF ER-ICM program theory.

**Methods:**

Descriptive statistics are used to describe HF ER-ICM participant characteristics. Focus group interviews, key informant interviews and fidelity assessments were conducted between November 2010 and January 2011, as part of the program implementation evaluation. In-depth qualitative interviews with HF ER-ICM participants and control group members were conducted between March 2010 and June 2011. All qualitative data were analysed using grounded theory methodology.

**Results:**

The target population had complex health and social service needs. The HF ER-ICM program enjoyed a high degree of fidelity to principles of both anti-racism/anti-oppression practice and Housing First and comprehensively addressed the housing, health and sociocultural needs of participants. Program providers reported congruence of these philosophies of practice, and program participants valued the program and its components.

**Conclusions:**

Adapting Housing First with anti-racism/anti-oppression principles offers a promising approach to serving the diverse needs of homeless people from ethno-racial groups and strengthening the service systems developed to support them. The use of fidelity and implementation evaluations can be helpful in supporting successful adaptations of programs and services.

**Trial registration:**

Current Controlled Trials ISRCTN42520374

## Background

### Homelessness and mental illness

Homelessness continues to be a complex social problem in Canada. Toronto, with a population exceeding 2.5 million residents, is home to the largest number of homeless people in Canada; an estimated 5,000 Toronto residents are homeless on any given night, and more than 28,000 individuals utilize homeless shelters each year [1,2].

Homelessness in Toronto is complicated by the ethnic diversity of the population and the large numbers of recent immigrants. The 2006 Canada census reports that half (47%) of Toronto residents are ethno-racial (see definition in Table 1[3,4]) and the population is comprised of more than 200 distinct ethnic ancestries [5]. Furthermore, half of all Toronto residents are immigrants to Canada, and 81% of new immigrants to Toronto between 2001 and 2006 were from visible minority groups [6]. The city of Toronto has identified ethno-racial and immigrant groups at high risk of homelessness [7]. Among the Toronto homeless population, a recent study of shelter or meal program users reported that almost half (45%) identified as belonging to a non-White ethnic group, most commonly Black (22%), and Aboriginal (9%) [8].

**Table 1:** Definitions of “Ethno-Racial” and “Racialized”

**Term**	**Definition**
Ethno-Racial	Includes persons who are racialized but not First Nations People. Also referred to as “people of colour” or “visible minorities” [4].
Racialized	The term racialized person/group refers to what was previously called "ethno-racial or people of colour" and First Nations People. The Ontario Human Rights Commission states: *“When it is necessary to describe people collectively, the term “racialized person” or “racialized group” is preferred over "racial minority,” "visible minority", "person of colour" or “non-White” as it expresses race as a social construct rather than as a description based on perceived biological traits*” [3,4].

Reports from Canada, US, UK and Australia suggest that immigrant and ethno-racial groups use mental health services less frequently compared to non-immigrants and experience significant barriers to care [9-14]. A local Community-University Research Alliance (CURA) project “Taking Culture Seriously in Community Mental Health” has similarly observed that specific cultural-linguistic groups experience numerous barriers to accessing culturally appropriate mental health services [15-17]. Such group-specific low use of mental health services is concerning because higher rates of mental health problems have been observed in immigrants, refugees and ethno-racial individuals in Canada and worldwide [14,18-23]. It is likely that a myriad of factors contribute to the reduced service use and access to services observed among immigrant and ethno-racial groups, including distinct perspectives about mental health and illness; culturally unique methods of expressing mental health problems; a desire for more culturally appropriate alternative interventions and treatment; perception of coercive treatment approaches; and a lack of understanding of the need for culturally appropriate approaches among programs and providers [24].

### At Home/Chez Soi in Toronto

In recent years, Housing First (HF) has emerged as the approach of choice to housing homeless individuals with mental illness [25-29]. The HF approach was first pioneered by *Pathways to Housing* (from now on, *Pathways*) in New York City in the 1990s [27]. Studies to date, although limited, have shown HF to produce favourable results in terms of reducing homelessness and decreasing the frequency of use of institutional services, including hospitals and correctional facilities [25-29]. Inspired by the success of the HF approach in the U.S., the Mental Health Commission of Canada has funded the At Home/Chez Soi Research Demonstration Project in Homelessness and Mental Health (AH/CS). AH/CS is a four-year pragmatic field trial testing the effectiveness and cost-effectiveness of HF interventions in addressing the needs of homeless individuals with mental illness. AH/CS is taking place in five cities across Canada (Moncton, Montreal, Toronto, Winnipeg and Vancouver) and is the largest controlled trial of HF worldwide. In each study site, a unique population or service context is examined. Given Toronto’s ethno-cultural diversity, the Toronto site research team, in consultation with local ethno-racial mental health service agencies, implemented an intervention targeting the needs of homeless people with mental illness from ethno-racial groups. Across Boundaries, an agency with extensive experience in anti-racism/anti-oppression (AR/AO) principles was selected to lead and implement the service model which combined HF principles with an AR/AO framework and practice.

### Anti-racism and anti-oppression

Both anti-racist and anti-oppressive principles are rooted in a commitment to social justice [30,31]. Oppression is an encompassing term that can be defined as a system of “*domination that denies individuals dignity, human rights, social resources and power*” (p.10) [32]. Anti-oppression, in the context of service provision in the fields of health and social services, can be seen as a theory that guides practitioners to address the issues of dignity, human rights, access to resources and power [33]. Racism is a form of oppression that includes a complex network of structures, actions and beliefs that result in the dominant racial group having an unequal distribution of resources, privilege, and power, at the expense of all other racial groups [34]. Anti-racist ideology focuses on transforming these unequal social relations and restoring power imbalances [32]. Like anti-racism, anti-oppression recognizes the existence of power imbalances and provides a framework on how to address them. The difference between anti-oppression and anti-racism lies in the fact the anti-oppression does not predefine oppression from a specific category or mechanism, whereas anti-racism takes race/racism as the point of entry in its analysis of oppression, power and privilege. The main principles of anti-oppressive and anti-racist service delivery have been outlined by Larson (2008) [35] and expanded by Corneau and Stergiopoulos (2012) [36] and include: empowerment, education, alliance building, language use, alternative healing strategies, advocacy, social justice/activism and fostering reflexivity (for detailed description see [36]).

### Housing First principles

The underlying philosophy of HF resides in the belief that housing is a human right; as such, in HF programs homeless individuals with mental illness are given access to permanent housing, similar to what is available to people who do not live with psychiatric or other disabilities [37,38]. Housing is provided in scattered site apartments, with only 10-20% of units in a given site dedicated to the program, to allow for community integration. Rent supplements are provided to clients to offset the cost of housing, and less than 30% of the client’s own income is used for housing costs [39]. Housing readiness does not need to be demonstrated prior to being housed and participants are not required to accept medical treatment or demonstrate sobriety prior to accessing housing [39,40]. Treatment and mental health services are part of the program and are available to participants, who see a case manager at least weekly. In the *Pathways* model, services are typically provided via an assertive community treatment (ACT) team [28]. An ICM model was chosen for the Toronto ethno-racial intervention, targeting homeless individuals with moderate needs not necessitating ACT. Other aspects of the *Pathways* approach were adopted as described above and are outlined in detail elsewhere [41,42].

### Housing First Ethno-Racial Intensive Case Management (HF ER-ICM): program description

The HF ER-ICM intervention combines AR/AO and HF frameworks of practice. Participants are offered rent supplements to access furnished scattered site apartments in the neighbourhood of their choice, in conjunction with intensive case management supports. Within this HF framework, commitment to AR/AO is manifest in program structures and management support, including hiring practices and regular staff training in AR/AO practices. Furthermore, services are delivered in a physical environment that is inclusive and welcoming of ethno-racial communities, offering linguistic and culturally accessible programming and services onsite. In addition to the services provided as part of intensive case management, the HF ER-ICM agency offers a variety of unique services, including art therapy, community kitchen, computer program, creative expression, life skills, music therapy, traditional Chinese medicine, yoga, as well as Women’s and Men’s support groups [4]. Staff explicitly address oppression and mental health together, adapting delivery of service to clients’ pace and recognizing variety in healing approaches. The program involves families and peer networks early in the recovery process [4].

Although there are no conditions of housing readiness or acceptance of psychiatric treatment in the HF ER-ICM program, study participants have two conditions to which they must agree: 1) a weekly face-to-face meeting with their case manager, typically in their residence, and 2) that <30% of their income will be used directly for rent. A maximum $600 monthly subsidy is paid directly to the landlord, which, in conjunction with <30% of the participants income (typically from social services), is used to cover the cost of rent. Participants are entitled to all rights and obligations as tenants under provincial legislation. The study budget also includes an allowance for furnishing and moving costs. [41].

### Objectives

This paper presents the findings of an early implementation and fidelity evaluation of a novel Housing First Ethno-Racial Intensive Case Management (HF ER-ICM) model, designed to meet the needs of homeless people with mental illness from ethno-racial groups.

In particular, the objectives of this paper are:

1) To document the successful recruitment of the ethno-racial sample and describe the target population;

2) To describe the fidelity of the HF ER-ICM program to both AR/AO and HF approaches and principles;

3) To report on program provider and program participant perspectives on the model; and

4) To identify challenges and facilitators of successful early implementation.

## Methods

### Ethics approval

This study was approved by the Research Ethics Board of St. Michael’s Hospital, and has been registered with the International Standard Randomized Control Trial Number Register (ISRCTN42520374).

### Study participants

Study participants for the HF ER-ICM had to meet all AH/CS study requirements. In addition, participants had to indicate membership in an ethno-racial group (see definition in Table 1[3,4]) and require a moderate level of service support which could be met by an intensive case management team (for further details, please see [41]). The AH/CS study protocol, and the Toronto specific study design, including eligibility criteria, has been described in detail elsewhere [41,42]. Participants in the treatment as usual (TAU) control group are still able to access the various available programs and services in the city of Toronto. TAU participants were provided with information about the availability of such services in the community and were directed to both mainstream and homeless-specific health services for care.

### Data collected

#### Quantitative data

Quantitative demographic data were collected from HF ER-ICM intervention and control participants at the screening and baseline interviews. Data collected at these interviews included demographic variables, mental illness diagnoses (including documented prior diagnoses and diagnoses made at study entry using the Mini International Neuropsychiatric Interview 6.0 [43]) and extent of community functioning (using the Multnomah Community Ability Scale [44]). See [41] for detailed description of all of the questionnaires used as part of the AH/CS study, and [42] for questionnaires used uniquely at the Toronto site.

#### Qualitative data

This paper draws on several lines of qualitative data, including the implementation and fidelity evaluation of the HF ER-ICM program, and the HF ER-ICM participant narratives.

#### Implementation evaluation

The implementation evaluation included interviews with key informants as well as focus groups with study participants and staff. Focus groups and key informant interviews conducted as part of the implementation evaluation took place between December 2010 and January 2011. In total, five focus groups were conducted: 1) staff from HF ER-ICM team (N = 7); 2) staff from Housing Team (N = 4); 3) members of consumer caucus (N = 12); 4) HF ER-ICM participants (N = 10); and 5) treatment as usual (control) participants (N = 5). Furthermore, five key informants were interviewed, including: 1) principle investigator from Toronto research team; 2) Toronto site study coordinator; 3) representative from the City of Toronto and its Housing Team; 4) director of the HF ER-ICM team; and 5) HF ER-ICM team lead. Participants who took part in the qualitative interviews for the implementation evaluation were identified by the Toronto Site project governance structure. Participants included stakeholders who played an integral role in the overall implementation of the HF ER-ICM program as well as participants and front line service providers.

#### Fidelity evaluation

A fidelity evaluation was conducted in November 2010 to assess the HF ER-ICM program fidelity to principles of both AR/AO and HF. Fidelity evaluations included observations of agency team meetings, interviews with frontline staff and managers and agency document and chart reviews.

Criteria for fidelity to AR/AO principles and a corresponding scale and method of assessment were developed by the research team, based on an examination of existing work regarding anti-racist and anti-oppressive principles and practice [32,36,45-52]. The process included a literature review, content expert interviews, and confirmatory methods examining the degree of consensus for the domains of the fidelity scale among those familiar with AR/AO theory and practice. Six key domains of fidelity to AR/AO principles were identified: 1) commitment to AR/AO; 2) human resource environment; 3) staff/program participant engagement and voice; 4) advocacy, community building and community engagement; 5) anti-racism frontline praxis; and 6) holistic treatment. Questions asked in the fidelity interviews focused on whether the program met the criteria within each of the domains. For example, in the domain of “commitment to AR/AO”, agency documents were reviewed and staff were asked “Does the agency have a formal commitment to Anti-Racism, whether in the form of a policy or mission, vision, value, or mandate statement?” In general, all questions asked of individuals participating in the fidelity evaluation pertained directly to the detailed criteria outlined for each of the domains of AR/AO fidelity in Table 2.

**Table 2:** Fidelity Assessment Tools and Summary of Findings from Anti-Racism/Anti-Oppression and Housing First Fidelity Evaluations at the Toronto site of the At Home/Chez Soi study

**I. FIDELITY TO ANTI-RACISM / ANTI-OPPRESSION**		
**FIDELITY ASSESSMENT TOOL**		**SUMMARY OF EVALUATION FINDINGS**
**DOMAINS**	**CRITERIA**	
**I. Commitment toAnti-Racism/ Anti-Oppression**	1. Agency has formalized its commitment to anti-racism and is committed to effective implementation of anti-racism.	▪Agency's commitment to anti-racism (AR) and anti-oppression (AO) principles was demonstrated via mission and policy statements, and mandates; management's accountability for and oversight of anti-racism activities; agency's participation, evaluation and formal commitment to addressing intersecting grounds of oppression.
**II. Human Resource Environment**	**II a) Anti-Racism Training and Professional Development**	**II a) Anti-Racism Training and Professional Development**
	1. Agency provides staff with educational activities in which anti-racism and anti-racism related issues (including anti-oppression, cultural competence, holistic theory and practice, etc.) are addressed and staff is required to have adequate training on these topics	▪All staff are well oriented to the agency’s AR/AO commitments and participate regularly in ongoing AR/AO training. A member of management staff oversees AR/AO training.
	**II b) Recruitment, Hiring and Retention**	**II b) Recruitment, Hiring and Retention**
	1. Agency is committed to hiring and retaining staff that are representative of the community served. Benchmarks include:	▪Recruitment and hiring of board, management and other staff was clearly informed by anti-racism competency considerations. Management and staff were reflective of the population served. Agency did well to track the ethno-racial composition of program participants and to target staff recruitment accordingly.
	a) Recruitment and hiring procedures that consider and assess anti-racism competency;	
	b) Frontline AND management staff are reflective of the community served;	
	c) Management and staff performance evaluations include items related to anti-racism; and	
	d) Staff satisfaction and retention level data disaggregated by racialized group are obtained and reviewed.	
**III. Staff/Program Participant Engagement and Voice**	1. Staff and program participants are able to have their concerns heard by management and influence direction-setting activities. Benchmarks include:	▪Agency offers numerous forums for staff and participants to have their concerns heard, including community meetings, staff retreats and staff meetings and there exists an informal culture of consultation and engagement in place, including an open-door management policy.
	a) An effective formal discrimination complaint mechanism is in place for staff;	
	b) An effective formal discrimination complaint mechanisms is in place for program participants;	
	c) Frontline staff have a voice in agency/program direction-setting; and	
	d) Program participants have a voice in agency/program-direction setting.	
**IV. Advocacy, Community Building & Community Engagement**	1. Agency is involved in advocacy-related and community building activities that serve the interests, health and wellbeing of its racialized program participants. Benchmarks include:	▪The agency evidences a keen appreciation of the importance of community engagement and advocacy, and engages in numerous advocacy-based initiatives and project partnerships. Agency staff have facilitated various community dialogues around health and broader issues in innovative ways that help counter stigmatization.
	a) The agency communicates and disseminates program/service information to racialized communities in the service areas;	
	b) The agency forms alliances and partnerships with anti-racism and/or racialized-specific organizations in the service area;	
	c) The agency engages in social justice advocacy to change or influence legislation or other intuitions’ policies that negatively impact the health and wellbeing of racialized program participants; and	
	d) The agency consults with racialized community members and organizations in the service area regarding the health-related concerns of its community	
**V. Anti-Racism Frontline Praxis**	1. Anti-Racism informs and is put into practice at the direct service level.	▪Case managers had an intuitive grasp of how AR/AO translates into their practice.
**VI. Holistic Treatment**	1. A holistic approach to health and wellness is adopted that informs program and service delivery. The program supports the following functions:	▪Client review during staff meetings and subsequent interviews with case managers and program manager demonstrated a very holistic approach to recovery planning, addressing broad social and cultural determinants of health. Numerous innovative alternative healing programs were offered by the agency (from yoga to drumming).
	a) Staff explore participants’ cultural views of wellness and illness;	
	b) Programs and services address and engage the families of service users, as desired;	
	c) A profile of social and cultural resources for various ethno-racial groups in the service area is maintained and made available to program participants (houses of worship, community-based organizations, etc.); and	
	d) Staff support participants in accessing alternative treatments (including those that address emotional, cultural and spiritual wellbeing), as desired.	
**II. FIDELITY TO HOUSING FIRST**		
**I. Housing Choice & Structure**	1. Program participants choose the location and other features of their housing (decorating, furnishing, etc.).	▪The program meets the housing choice and structure domain with the highest standards for all criteria except for housing availability.
	2. Program participants are moved quickly into housing of their choosing once they acquire housing subsidy.	
	3. Housing tenure is assumed to be permanent with no actual or expected time limits other than those on standard occupancy agreement.	▪With respect to housing availability, about two-thirds (67%) of the participants moved into the housing unit of their choosing within 6 weeks of receiving housing subsidy. Housing delays occurred for some participants due to trying to find the right housing that fits the participant’s preferences. Participants have an extraordinary amount of choice in location and sometimes the search for a “perfect match” for housing can significantly delay move-in and places strain on team resource.
	4. Housing is affordable (<30% of income).	
	5. Housing is integrated in scatter-site private market housing which is otherwise available to individuals who do not have psychiatric or other disabilities (also, <20% of units in a building are leased out by the program).	
	6. Housing is private (no expectation to share living spaces).	
**II. Separation of Housing & Services**	1. Participants are not required to demonstrate housing readiness prior to access to housing units.	▪The ER-ICM program meets the separation of housing and services domain with the highest standards for all criteria.
	2. Tenancy is not linked in any way to adherence to treatment or service provisions.	
	3. Program participants have legal rights to unit as per lease or occupancy agreement, with no special provisions added.	
	4. Participants have access to a new housing unit if they lose their housing access.	
	5. Participants continue to receive services even in the event of housing loss (eviction, inpatient treatment, etc.).	
	6. Social and clinical service providers are not located at participants’ residence.	
	7. Social and clinical service providers are mobile and can deliver services at locations which the participants choose.	
**III. Service Philosophy**	1. Participants choose the type, sequence and intensity of services on an ongoing basis.	▪The ER-ICM program meets the service philosophy domain criteria for all areas with the exception of harm reduction approach, motivational interviewing and person-centered planning, where additional training would be helpful.
	2. Participants with psychiatric disabilities are not required to participate in treatment or take medication.	
	3. Participants with substance use disorders are not required to participate in treatment.	
	4. Program utilizes a harm reduction approach.	
	5. Program staff use principles of motivational interviewing in all interactions with participants.	
	6. Program staff use an array of techniques to engage difficult-to-engage consumers including a) motivational interventions; b) therapeutic limit-setting interventions. The program also has a process for identifying the need for assertive engagement, including measuring effectiveness of assertive engagement techniques and modifying these approaches as necessary.	
	7. Program does not engage in coercive activities towards participants (e.g. leveraging housing or services to promote adherence to clinical provisions OR having excessive intrusive surveillance with participants).	
	8. Program engages in person-centered planning, including a) development of formative treatment plan; b) conducting regular scheduled treatment planning meetings; c) actual practices reflect strengths and resources identified in the assessment.	
	9. Program systematically delivers specific interventions to address a range of life areas (e.g. physical health, employment, education, social support, recreation, etc.).	
	10. Program increases participants’ independence and self-determination (by providing choices as much as possible).	
**IV. Service Array**	1. Program offers housing support services to help participants retain housing.^1^	▪The ER-ICM program meets the service array domain criteria for providing housing support and social integration services, brokering psychiatric services, and involvement in inpatient treatment admission.
	Program provides active referrals and conducts follow-up for the provision of:	
	2. Psychiatric services^2^	
	3. Substance abuse services^2^	
	4. Employment and education services^2^	
	5. Nursing/medical services^2^	
	6. Program provides services supporting social integration, including a) facilitating access to and helping participants develop valued social roles and networks within and outside the program; b) helping participants develop social competencies to successfully negotiate social relationships, c) enhancing citizenship and participation in social and political venues.	
	7. Program responds to psychiatric or other crises 24 hours a day.	
	8. Program is involved in inpatient treatment admission.^3^	
**V. Program Structure**	1. Program has priority enrollment for individuals with obstacles to housing stability (homelessness, severe mental illness, substance use)	▪The ER-ICM program was able to meet almost all of the criteria outlined in the Program Structure domain. Development of new strategies for improving contact with difficult to see participants will help ensure ongoing contact. Establishing new opportunities for participant involvement in the program would increase their representation in program operations.
	2. Program has a minimal threshold of non-treatment related contact with participants	
	3. Program has a low participant to staff ratio (20 or fewer participants per 1 full time staff)	
	4. Program has a team approach	
	5. Program has frequent meetings where program staff plan and review services for participants	
	6. Program has weekly meeting/case review.^4^	
	7. Participants are represented in program operation and have input into policy (including roles on committees and governing bodies as well as peer advocates)	

^1^ Housing support services including services such as neighbourhood orientation, landlord/neighbour relations, property management services, assistance with rent payment or subsidy assistance, utility setup, co-signing of leases, budgeting and shopping.

^2^ The criteria for successfully brokering each service includes: 1) The program has established formal and informal links with several providers; 2) The program assesses participants in order to match participant needs and preferences to providers; 3) The program assists participants in locating, obtaining and directly introducing participants to providers; and 4) The program conducts follow-up, including communication with other providers regarding services on a regular basis and coordinating care.

^3^ The program works with inpatient staff to ensure proper discharge with the following steps: 1) program initiates admissions as necessary; 2) program consults with inpatient staff regarding need for admissions; 3) program consults with inpatient staff regarding participant’s treatment; 4) program consults with inpatient staff regarding discharge planning and 5) program is aware of participant’s discharge from treatment.

^4^ Weekly meeting/case review should serve the following functions: 1) conduct a brief but clinically relevant review of half the caseload; 2) discuss participants with high priority emerging issues in depth to collectively identify potentially effective strategies’ and approaches; 3) identify new resources within and outside the program for staff and participants; and 4) discuss program-related issues such as scheduling, policies, procedures, etc.

Fidelity to HF principles was assessed using criteria previously outlined by the *Pathways* model [39] but also included elements from other ACT and supportive housing scales and measures [53-57]. The key five domains that comprised the HF fidelity assessment tool were based on the program fidelity essential ingredients checklist provided by *Pathways Housing First*[39,58] and included the following: 1) housing choice and structure; 2) separation of housing and services; 3) service philosophy; 4) service array; and 5) program structure. Table 2 outlines the HF fidelity domains, the detailed criteria for each of the five domains of HF fidelity, and provides a summary of the HF fidelity evaluation findings.

Fidelity to AR/AO was assessed by members of the research team and AR/AO experts who were independent to the study. The program’s fidelity to HF principles was assessed by an independent committee that was comprised of members of the *Pathways Housing First* program.

#### Participant narratives

A subset of HF ER-ICM participants participated in in-depth qualitative interviews between March 2010 and June 2011. Two types of interviews were conducted. The first set of interviews included participants from all three study intervention arms and examined the participants’experiences of homelessness and mental illness and identified particularly memorable life events (including any high point, low point or turning point stories) in a 90 minute interview. A total of 10 randomly selected participants from the HF ER-ICM intervention participated in this first set of qualitative interviews.

A second set of participant narratives included participants who indicated ethno-racial ethnicity and evaluated participant coping strategies along the intersecting dimensions of homelessness, mental illness, race and gender. A subset of 40 ethno-racial participants, including 15 from the HF ER-ICM intervention group and 12 from the control group participated in these interviews. Participants were part of a convenience sample of individuals who were reflective and articulate and were identified as such by the research team. Participants were asked a number of questions about their experiences with homelessness, mental illness and racial/cultural discrimination in interviews that lasted between 1 to 2 hours.

### Data analysis

#### Quantitative data

Frequencies, percentages, means and standard deviations were reported for questionnaires, where appropriate. All analyses were preformed with IBM SPSS 20 (IBM, Chicago, Illinois).

#### Qualitative data

Interview and focus group transcripts were analyzed using grounded theory methodology. The grounded theory analysis employs inductive strategies, systematic coding and comparative analysis procedures to analyze “*individual cases, incidents, or experiences and develop progressively more abstract conceptual categories to synthesize, to explain and to understand…data, and to identify patterned relationships within it*” (p. 497) [59]. Transcripts were coded by the study interviewers. Field notes were recorded by the interviewer upon completion of each interview and focus group; these notes served to support and elaborate upon the themes from the key informant interview and focus group data. Line-by-line coding was used, which involves reading through sections of transcripts of interviews carefully to identify key concepts [60]. As reliability checks during this process, the interviewers double-coded several transcripts, and compared their findings. Once consensus was achieved, interviewers proceeded to code the remaining transcripts. At this stage, a larger group of qualitative researchers from the team discussed the categories and collectively reduced the categories to a smaller set of higher-level themes.

## Results

### Target population

Table 3 summarizes the key demographic characteristics of HF ER-ICM program participants. In total, 204 participants were eligible for the HF ER-ICM program, of which 102 were randomized to the intervention, while the remaining 102 were randomized to the control group.

**Table 3:** HF ER-ICM Participant Demographics

	**TOTAL SAMPLE**^1^	
	**N=204**	**% Missing**
	**Mean (±SD) or N(%)**	
**DEMOGRAPHICS**		
Age (years)	38.6 ± 12.1	0 (0.0)
Gender		0 (0.0)
Female	70 (34.3)	
Male	130 (63.7)	
Other^2^	4 (2.0)	
Country of birth		0 (0.0)
Afghanistan	4 (2.0)	
Canada	50 (24.0)	
China	5 (2.5)	
Ethiopia	9 (4.4)	
Ghana	5 (2.5)	
Haiti	3 (1.5)	
Hong Kong	3 (1.5)	
India	3 (1.5)	
Iran	6 (2.9)	
Jamaica	26 (12.7)	
Mexico	3 (1.5)	
Philippines	4 (2.0)	
Somalia	8 (3.9)	
Sri Lanka	10 (4.9)	
St. Vincent and the Grenadines	4 (2.0)	
Trinidad and Tobago	6 (2.9)	
Vietnam	4 (2.0)	
Other^4^	51(25.0)	
Native language		0 (0.0)
English	89 (43.6)	
French	7 (3.4)	
Other	108 (52.9)	
Ethnic or cultural identity		0 (0.0)
Black - Africa	40 (19.6)	
Black - Canada	25 (12.3)	
Black - Caribbean	45 (22.1)	
East Asian	12 (5.9)	
Indian-Caribbean	2 (1.0)	
Latin American	10 (4.9)	
Middle Eastern	15 (7.4)	
Mixed Background	22 (10.8)	
South Asian	21 (10.3)	
Southeast Asian	12 (5.9)	
**HOMELESSNESS**		
Current housing status		0 (0.0)
Absolutely homeless	185 (90.7)	
Precariously housed	19 (9.3)	
Total length of homelessness during lifetime (years)	3.48 ± 4.64	5 (2.5)
Longest period of homelessness (years)	1.84 ± 2.58	1 (0.4)
**EDUCATION AND EMPLOYMENT**		
Education history		0 (0.0)
Didn’t complete high school	87 (42.6)	
Completed high school	40 (19.6)	
Completed at least some post-secondary	77 (37.7)	
Currently employed	10 (4.9)	
**MENTAL AND PHYSICAL HEALTH**		
MCAS score	65.5 ± 3.15	0 (0.0)
Written documentation of a mental disorder	42 (20.6)	2 (1.0)
MINI Results		0 (0.0)
Current Depressive Episode	81 (39.7)	
Current Manic Episode or Hypomanic Episode	15 (7.4)	
Current Post-Traumatic Stress Disorder (PTSD)	48 (23.5)	
Current Panic Disorder	36 (17.6)	
Current Mood Disorder with Psychotic Features	48 (23.5)	
Current Psychotic Disorder	74 (36.3)	
Current Alcohol Dependence	39 (19.1)	
Current Substance Dependence	50 (24.5)	
Current Alcohol Abuse	31 (15.2)	
Current Substance Abuse	19 (9.3)	
Current Suicidality		
*Low*	71 (34.8)	
*Moderate*	44 (21.6)	
*High*	15 (7.4)	

^1^ For the total sample, percentages shown were calculated as proportion of the total sample (N = 575) and therefore the column totals for each variables will not add up to 100% if data was missing (see adjacent column with N and% Missing in total sample).

^2^ “Other” category includes individuals who identify as Transgendered, Transsexual or Other.

^3^ The “Other” category for country of birth includes individuals born in countries not listed. These individuals were born in countries representing the following broad geographic regions: Africa, N=21; Caribbean, N=5; Central and South America, N=5; East Asia, N=5; Europe, N=4; Middle East, N=4; South Asia, N=4; and Southeast Asia, N=3.

The sample was mostly male (64%), with a mean age of 38.6 ± 12.1 years, and an average length of lifetime homelessness of 3.48 ± 4.64 years. More than half (53%) of participants reported a native language other than English or French. Almost half (44%) reported their first language as English, but only a quarter (24%) of the sample was born in Canada. Participants reported various cultural identities, although most self-identified as Black (54%), either from the Caribbean region (22%), Africa (20%) or Canada (12%). Other groups included South Asian (10%), Middle Eastern (7%), East Asian (6%), Southeast Asian (6%), Latin American (5%), and Indian-Caribbean (1%). About a tenth (11%) of the sample comprised of individuals of mixed background.

Results from the MINI neuropsychiatric interview indicated that many participants suffered from suicidality (64%), depression (40%), psychotic disorder (36%), substance dependence (25%), mood disorder with psychotic features (24%), post-traumatic stress disorder (PTSD) (24%), alcohol dependence (19%) panic disorder (18%), alcohol abuse (15%), substance abuse (9%), and bipolar illness (7%).

Most participants were referred from the shelter system (50%), while mental health services (12%), drop-in centres (11%) and hospitals (9%) were the other main referral sources.

### Fidelity evaluation

A summary of the HF ER-ICM program fidelity evaluation findings, are presented in Table 2 and summarized below.

### Fidelity to AR/AO

The ER-ICM program demonstrated high fidelity to AR/AO principles in all of the domains examined in the fidelity evaluation (commitment to AR/AO; human resource environment; staff/program participant engagement and voice; advocacy, community building and community engagement; anti-racism frontline praxis; holistic treatment). The agency showed a strong commitment to anti-racist and anti-oppressive principles, including formally recognizing this commitment in the agency’s mission and policy statements as well as management’s accountability and oversight of anti-racism activities. All staff were familiar with AR/AO principles and participated in regular AR/AO training. Hiring practices and staff recruitment were informed by anti-racism competency at all levels (board, management and service providers), and both management and staff were representative of the population served. Numerous forums were available to both staff and participants for engagement, consultation, and voicing of opinions, including community meetings, staff retreats, and staff meeting. Advocacy, community building and community engagement were important to the agency and numerous advocacy-based initiatives and project partnerships had been undertaken, including community dialogues to identify unmet needs and community driven approaches to meeting them. The holistic treatment was one of the HF ER-ICM program’s greatest strengths and was demonstrated by numerous and innovative healing programs offered at the agency (from yoga to drumming), addressing social and cultural determinants of health. Two potential areas of improvement included developing an anti-racist strategic plan of action with clear performance goals and measures, and secondly, further developing frontline service delivery by better documenting implementation of anti-racism theory in daily service delivery.

### Fidelity to Housing First

In addition to adherence to AR/AO principles, the ER-ICM program demonstrated a high degree of fidelity to HF principles in all of the domains examined in the fidelity evaluation (housing choice and structure; separation of housing and services; service philosophy; service array; program structure). The program encouraged participant choice, ensuring that the location and home itself were the right fit for the participants. The principles of separation of housing and services were also upheld and participants receive a standard lease with all the rights and responsibilities of tenancy. Agency staff were deeply aware of participant needs and provided participant-driven services from a recovery-oriented and harm reduction framework. No treatment requirements were necessary for participants to continue in the program. The team provided comprehensive supports, including an in-house psychiatrist, linking participants to medical service providers and other services as needed. Finally, the HF ER-ICM team employed a strong program structure even though they used an individual caseload approach. Team meetings were held regularly and allowed staff to be familiar with other cases and discuss best strategies for shared challenges. Two areas identified for improvement included a need for additional training in harm reduction and motivational interviewing (including how to implement both in daily practice) and expanding links with brokerages services, particularly in the areas of employment and education, substance use treatment, and nursing/medical services.

### Understanding program ingredients and program theory: service provider and participant perspectives

Among HF ER-ICM service providers, both key informants and focus group participants noted congruence between HF and AR/AO approaches to practice. Specifically, a commitment to client-driven recovery and harm reduction was noted to be an underlying value that informs both HF and AR/AO models. As one key informant noted, The need for both staff and clients to engage and talk about issues of oppression and racism was identified as an important component of the program, allowing for focused attention to issues of power. Using techniques of motivational interviewing, and by sharing their own past experiences, staff encouraged clients to speak explicitly about discrimination, and helped them to name racism or oppression where they may have encountered it. One staff focus group participant described this as, *“…giving [clients] the opportunity… letting them know they can talk about [racism and discrimination].”* This approach allowed for frequent debriefing and provided a formal complaint mechanisms for both staff and participants through which they discussed their experiences of discrimination. As one key informant stated,

“I know that recovery is a big part of this model as well … recovery really aligns very well to the anti-oppression framework and … we talk about racism and discrimination and name it which talks about choice and talks about people’s hope and recovery.”

“[Staff] need to be able to speak to [issues of race and oppression] and if the organization support isn’t there and the culture isn’t there, then you’re kind of missing the boat a little bit…So, if you’re not able to name the issue and if you can’t chat with the client about their experiences then you are not really addressing it…”

“I get a call from the worker saying ‘Oh my God, we had such a bad experience, I think the client is going to go to do something bad today… the superintendent treated us really badly, it was a white women, she wanted to know, are you on drugs … it’s people like you that I have a problem renting to, I am going to check your criminal record, I am going to check your financial records’ …it became a little bit of a race issue… I said you know, we will put it writing and make a complaint…I will forward it to the housing team and the staff also… she (the worker) was crying a lot on the phone, she’s never been treated like, she’s seen clients get treated like this but now she said both of us got treated really bad and so I was able to say what do you think that was, and [she was] like that was racism, they just didn’t understand why we you know, we’re making a big deal.”

Providing a service environment that is welcoming and inclusive to racialized communities was also regarded as necessary in order to address experiences of exclusion and enhance notions of citizenship or belonging amongst these groups. As one key informant noted, this was achieved, in part, by offering linguistically and culturally accessible programs and services within a “*safe and open*” space, where representation of the diversity of ethno-racial groups amongst service users is displayed.

Key informants also described the importance of agency involvement in advocacy for system-level changes to address the needs of racialized groups. Anti-racist approaches and analyses were seen to highlight inequitable inter-group social dynamics, and support empowerment processes that are critical to health and well-being. Such an approach to health requires that communities are engaged and mobilized, and that systemic issues beyond the agency’s immediate control are addressed through collaborative advocacy efforts.

Finally, the holistic approach to service delivery encouraged participants to access a range of treatment and support options that address both social and cultural determinants of health. For example, clients were able to cook and eat on-site in a community kitchen, which fosters a community environment and facilitates learning, skill development, sharing of stress and healing. As one key informant described,

“…we have a space that [clients] can come to…the best part is they’re able to come to the centre because it’s a drop in…where people can eat, people when they eat they feel better…the drop in really works, the drop in, people just dropping in and hanging out, watching some TV, playing this, just talking that’s what making this work.”

Service providers suggested that the AR/AO approach fosters improvements in health and mental health through a number of pathways, including empathic validation, empowerment, role modelling, and a corrective experience of inclusion, which in turn are hypothesized to facilitate participation in culturally relevant treatment of physical, mental health and addiction problems and support client recovery [36].

### Participant views

Participant feedback regarding the HF ER-ICM program was favourable. A participant focus group indicated that the HF ER-ICM program was highly valued, and that case managers were supporting participants to secure and maintain housing that meets their needs. Furthermore, HF ER-ICM case managers were thought to help advance recovery goals within client driven, holistic framework. As one focus group member noted,

“Oh, listen it’s excellent, believe me this…my worker [Name removed] he, he, he’s been there with me 24/7 I could say because you know, he keeps checking on me, he keeps calling me to see how I am doing, if I need anything, if I need to go anywhere, if I need assistance in anything, he is right there you know, and, he took me to the place there and when I saw it, I just get down on my knees there and started to pray, and thank God for it”

Another focus group member described their HF ER-ICM worker,

“She’s, she’s a very good worker…and that…never argue, make, make like a, a to do list, a calendar of what I have to do during the month, or what’s my certain appointments, cause sometimes I forget, so… it’s like I say, like… a lot of things… I’m achieving too, on top of the things that I want… to say that I have a lot of help there.”

Some participants further reflected on the unique opportunities for holistic treatment in the HF ER-ICM program:

“I get to know the program here, and I feel more comfortable, I feel more, more, more secure. My case worker counsel me when I’m going through depression, the hard times, she tell me things going to be okay… most of the time like I come here we have fun, we have like, like classes, we have like music therapy, art therapy. Computer class and we have like…creative expression”

### Challenges and facilitators of program implementation

One of the key challenges during implementation of the HF ER-ICM program was meeting the cultural and linguistic requirements of a highly diverse group of participants. Despite the cultural diversity and experience of HF ER-ICM case managers, the team encountered difficulties providing language and cultural backgrounds to suit the needs of all ethno-racial clients in their caseload. In particular, the case managers had typically worked with the South Asian and African-Caribbean communities in Toronto, but needed to accommodate Chinese and Korean clients. As one key informant described,

"“We have clients that speak different languages…in my team I don’t have a Korean-speaking member, so we have a few clients now… we have to tap into the translation services outside of what we already have.”"

The HF ER-ICM agency was able to meet these challenges by hiring peer workers, seeking out staff who better reflect the diverse make up of the participants, and by employing translational or linguistic services from other agencies. Over time, the team was able to successfully recruit diverse ethno-racial staff to assist in appropriately addressing participants’ needs.

Several elements facilitated the successful implementation of the HF ER-ICM model. Firstly, the extensive experience of the HF ER-ICM agency with AR/AO practice allowed for the successful implementation of this novel program with relative ease. Agency support for both supervisory and administrative staff with relevant AR/AO experience enhanced the likelihood of the accessibility and use of AR/AO practice [4]. As one key informant summarized,

“Staff need the culture or the freedom to speak about the issues of the racialized clients… managers or the supervisors need to have that analysis and that understanding so then they are able to help support the staff in terms of how they work with the client. … [If not] then the staff get to a point where they stop raising the issue, and they do the best they can but then it becomes that one off piece, right? It doesn’t become organizational support to racialized clients.”

The hiring practices of the HF ER-ICM agency were also identified as a key element; hiring of staff members from ethno-racial backgrounds that mirror the clients’ own background provided role models for the clients, and increased knowledge about the lived experiences of individuals in particularly communities, in addition to allowing for better alliance between worker and client.

With regards to the Housing First program components, in addition to extensive training and technical assistance around HF principles, the relationships between project partners have been important in the facilitation of the project, which fostered a collaborative learning and problem-solving environment. Furthermore, the strong partnerships between the housing team (Housing Connections) with landlord associations were invaluable to the housing process.

## Discussion

The HF ER-ICM program serves ethno-racial individuals with a history of homelessness and a significant burden of mental health and substance misuse problems, similar to those reported in other Housing First interventions. In addition to these challenges, HF ER-ICM program participants face additional challenges, three quarters being immigrants to Canada and half having a native language other than English or French. Participant demographics highlight the importance of adapting HF to better serve specific subpopulations and service delivery contexts. A union of AR/AO frameworks of practice with HF principles is one such adaptation that is being studied as part of the AH/CS project. The resulting combined treatment model addresses many of the unique needs faced by homeless individuals from ethno-racial groups, while still providing the core components of a HF intervention.

Although developed for different client groups, both AR/AO and HF principles share elements of client empowerment and choice, with AR/AO practices having an additional unique focus on family and community of origin, holistic treatment and explicit discussion of racism, discrimination and power inequities.

Client choice and empowerment are important elements in both AR/AO and HF approaches. AR/AO practice places strong emphasis on participant choice and clients are allowed to work as a team with their worker to select the program of action that best suits their own needs, influenced by their personal history, knowledge and experiences [61]. In the HF program, choice is a fundamental and offered to the client throughout the program, including in the choice of housing and the choice of acceptance of treatment [27]. Choice allows the consumer to feel a sense of personal control or mastery, particularly over the forces that are important in their lives [62].

Similarly, personal empowerment is the consumer’s perception of control over their life circumstances [63]. In AR/AO practice, empowerment is one of the key guiding principles, allowing the consumer to regain control that may have been lost due to the power struggles that exist in society [36]. Empowerment in AR/AO practice is achieved by involving consumers in the decision making processes, and by validating their experiences, belief systems and self [36]. In HF, empowerment is observed in the decisions and choices the client makes with regards to their housing and service provision. Using a strengths-based, recovery-oriented approach, coupled with motivational interviewing, clients are empowered to work on their recovery goals.

In AR/AO models, client empowerment and choice are further manifest in the variety of healing approaches available to clients, based on their traditions and belief systems. Beyond traditional biomedical practices, holistic treatment can include Chinese traditional medicine, Indian Ayurveda, African approaches, and yoga and other treatment approaches that approach healing from different worldviews [36].

Another key element of empowerment in anti-oppressive practice involves mobilizing the consumer’s strengths and networks of family and community of origin support which will allow them to gain more control over their lives [32,36]. Several studies have stressed the importance of family supports in decreasing the risk of homelessness in individuals with mental illness [50-53]. Family members can provide much care and support, often acting as the informal caregivers and providing emotional support as well as helping with securing housing and adequate treatment [64].

Also unique to the AR/AO approach is the emphasis on the wellcomeness of the physical environment, and the explicit discussion of experiences of racism and/or discrimination, as outlined in program descriptions. Brown (2003) proposes that discrimination and racism can produce mental health problems via their ability to generate stressful circumstances and emotional distress [65]. HF ER-ICM service providers teach participants about the pervasive nature of racism and how it can negatively affect all aspects of their life, including their mental health. Participants can address the feelings brought up by their own personal experiences of discrimination and racism by being given a platform where they can vocalize these experiences, and service providers act as role models offering empathic validation, problem solving strategies, and a corrective experiences of wellcomeness and inclusion.

The development and successful implementation of the HF ER-ICM program holds promise in offering homeless people with mental illness from ethno-racial groups the services and supports needed to aid their recovery. The strength of this approach lies in that it can be applied broadly, to multiple ethno-racial groups. As with all programs, the HF ER-ICM program model has some limitations. First, while it is possible to serve individuals from a wide variety of ethno-racial groups, it is possible that individuals from poorly represented ethno-racial groups will continue to face barriers to care due to shortages in case managers and peer support from these groups, and limited staff knowledge or expertise about their community of origin. Furthermore, AR/AO approaches may not find support in all social settings and service delivery contexts. Last, but not least, although HF holds great promise as an intervention for homeless people with mental illness, a small but significant number of homeless individuals cannot successfully live independently in the community as they require a higher level of support.

This study has some limitations. This implementation evaluation was conducted at an early stage of the program, before service providers and participants had the advantage of a long exposure to the intervention. It is possible that a later implementation evaluation would highlight additional critical elements, barriers and facilitators to implementation. Furthermore, the number of program participants interviewed was small, potentially limiting generalizability. On-going evaluation of the HF ER-ICM program, however, will allow us to address these limitations at a later stage.

## Conclusions

Housing First approaches can be enhanced to better meet the needs of specific populations and service contexts. Such adaptations can inform service delivery to vulnerable people that traditional services and supports have failed to serve adequately. When serving homeless people with mental illness from ethno-racial groups, combining HF principles with an AR/AO framework of practice is a promising approach.

## Competing interests

The authors declare that they have no competing interests.

## Authors’ contributions

VS is a Principal Investigator on the grant and oversaw all research activities, analysed the qualitative data, contributed to the writing of the manuscript and read all drafts of the manuscript. PO is a Principle Investigator on the grant and contributed to writing the manuscript. AG analysed the quantitative data, interpreted the findings and drafted the manuscript. JJ analysed the qualitative data and conducted the Implementation Evaluation at the Toronto site. SC conducted the literature searches on anti-oppression and anti-racism and mental health among racialized populations and contributed to the writing on these pieces. AS contributed to program development, fidelity evaluation and writing the manuscript. SWH is a Principle Investigator on the grant and contributed to revising the manuscript. All authors read and approved the final manuscript.

## Pre-publication history

The pre-publication history for this paper can be accessed here:

http://www.biomedcentral.com/1472-6963/12/345/prepub
